# Combining guilt-by-association and guilt-by-profiling to predict *Saccharomyces cerevisiae *gene function

**DOI:** 10.1186/gb-2008-9-s1-s7

**Published:** 2008-06-27

**Authors:** Weidong Tian, Lan V Zhang, Murat Taşan, Francis D Gibbons, Oliver D King, Julie Park, Zeba Wunderlich, J Michael Cherry, Frederick P Roth

**Affiliations:** 1 Department of Biological Chemistry and Molecular Pharmacology, Harvard Medical School, Longwood Avenue, Boston, Massachusetts 02115, USA; 2 Department of Genetics, School of Medicine, Stanford University, Stanford, California 94305-5120, USA; 3 Center for Cancer Systems Biology (CCSB), Dana-Farber Cancer Institute, Jimmy Fund Way, Boston, Massachusetts 02115, USA; 4 McKinsey and Company, Hansen Way, Palo Alto, California 94304, USA; 5 Merrimack Pharmaceuticals, Kendall Square, Cambridge, Massachusetts 02139, USA; 6 Boston Biomedical Research Institute (BBRI), Grove St., Watertown, Massachusetts 02472, USA; 7 Massachusetts Institute of Technology, Massachusetts Ave, Cambridge, Massachusetts 02139, USA

## Abstract

**Background:**

Learning the function of genes is a major goal of computational genomics. Methods for inferring gene function have typically fallen into two categories: 'guilt-by-profiling', which exploits correlation between function and other gene characteristics; and 'guilt-by-association', which transfers function from one gene to another via biological relationships.

**Results:**

We have developed a strategy ('*Funckenstein*') that performs guilt-by-profiling and guilt-by-association and combines the results. Using a benchmark set of functional categories and input data for protein-coding genes in *Saccharomyces cerevisiae*, *Funckenstein *was compared with a previous combined strategy. Subsequently, we applied *Funckenstein *to 2,455 Gene Ontology terms. In the process, we developed 2,455 guilt-by-profiling classifiers based on 8,848 gene characteristics and 12 functional linkage graphs based on 23 biological relationships.

**Conclusion:**

*Funckenstein *outperforms a previous combined strategy using a common benchmark dataset. The combination of 'guilt-by-profiling' and 'guilt-by-association' gave significant improvement over the component classifiers, showing the greatest synergy for the most specific functions. Performance was evaluated by cross-validation and by literature examination of the top-scoring novel predictions. These quantitative predictions should help prioritize experimental study of yeast gene functions.

## Introduction

The rapid development of high-throughput technologies has made it possible to study the properties and relationships of thousands of genes in parallel [1-6]. A current challenge in genomic analysis is to combine this increasingly rich trove of evidence to infer biological function. The sheer volume of information makes manual human evaluation impracticable. Therefore, it is urgently necessary to develop improved automated algorithms to assist and accelerate the process of functional annotation.

The transfer of function annotation from one gene to another via biological relationships ('guilt-by-association') has been widely used, especially on the basis of homology relationships. However, accurate functional annotation via homology often requires high sequence similarity between homologous proteins [7], and many proteins do not have homologs with known function. Function may also be assigned to a gene based on a profile of its characteristics ('guilt-by-profiling'). Sequence-based guilt-by-profiling methods, for example, assign functions based on matches to motifs derived from multiple sequence alignments [8-11].

Sequence-based relationships and characteristics are better suited to predict catalytic activity or structural role rather than involvement in a biological process, for example, osmotic stress response. Biological relationships other than homology have proven useful in guilt-by-association studies, including protein-protein physical interactions [12], genetic interactions derived from examination of double-perturbation and phenotyping experiments [13], correlated gene expression [14], or correlated phylogenetic profiles [15]. Algorithms to predict function from interactions have evolved from simple inspection of neighborhood interactions [12,16] to consider the global structure of the interaction network [17,18].

Beyond sequence patterns, a wide variety of biological characteristics has proven useful in guilt-by-profiling function prediction, including phenotype [19,20], subcellular localization [21], gene chromosome neighborhood [22], previously known functional annotations [23], and membership within gene expression clusters [24,25] or protein complexes [5].

Many guilt-by-profiling and guilt-by-association efforts have been reported. Some guilt-by-profiling studies have exploited gene-gene relationships by transforming them into gene characteristics [14], and some guilt-by-association studies have exploited gene characteristics by treating shared annotation with a gene characteristic as a gene-gene relationship. Only a few attempts have been made to integrate both types of inference. In particular, Deng and coworkers [26] inferred gene function by exploiting both gene-gene relationships and gene characteristics. This approach, discussed further below, used protein pattern annotation and protein complex information as gene characteristics, and also exploited protein interaction, genetic interaction, and expression correlation relationships.

Here, we introduce *Funckenstein*, a new method combining both guilt-by-profiling and guilt-by-association approaches to predict protein function. Initially, *Funckenstein *uses separate classifiers for guilt-by-profiling and guilt-by-association. It can integrate a very large number of gene characteristics and gene-gene relationships to infer functions, thanks to the scalability of its component classifiers. *Funckenstein *then combines prediction results using logistic regression optimized for precision versus recall performance, achieving a better performance than either approach alone.

Here we apply *Funckenstein *to a benchmark of integrated *Saccharomyces cerevisiae *genomic data used previously to predict broad gene functions, and show that *Funckenstein *achieves higher precision at all levels of recall. Because predictions of more specific gene functions are generally more useful to experimental biologists, we also apply *Funckenstein *to score 2,455 Gene Ontology (GO) terms for all protein-coding genes in *S*. *cerevisiae*. The results are assessed by cross-validation and by evaluation of top predictions by an expert curator from the *Saccharomyces *Genome Database (SGD). Together, the results show that *Funckenstein *achieves high precision in cross-validation and in the prediction of novel functions.

## Results

### A method combining guilt-by-profiling with guilt-by-association

We have developed an algorithm (*Funckenstein*) that combines both guilt-by-profiling and guilt-by-association to make gene function predictions. Guilt-by-profiling was performed using the random forest (RF) method [27]. However, for comparison we also performed guilt-by-profiling using a probabilistic decision tree (PDT) method [28] with an early-stopping criterion to limit over-fitting. Guilt-by-association was performed in two steps: first, generate a functional linkage (FL) graph [29], that is, a graph with edge weights reflecting the probability that two genes share a sufficiently specific GO term; and second, use the FL graph to assign a score to each (candidate gene, GO term) combination based on the weight of links between the candidate gene and genes currently assigned to that GO term. The RF and FL predictions were then combined via a logistic regression model that optimizes precision versus recall performance. A full description of the algorithm and optimization of its parameters may be found in Materials and methods.

### Performance evaluation using a previous benchmark

A combined approach was applied previously to predict *S*. *cerevisiae *gene functions. Specifically, a Markov random field (MRF) guilt-by-association approach integrated information from protein interactions, genetic interactions, and gene expression correlation [26]. This approach also employed guilt-by-profiling, in that a gene-specific prior was calculated from Pfam protein sequence patterns [8] and protein complex membership using a naïve Bayes (NB) method [30]. We will refer to this combined approach as 'MRF-NB'.

MRF-NB was applied previously to protein-coding *S*. *cerevisiae *genes to predict a small set of functional categories derived from literature annotation by the Munich Information Center for Protein Sequences (MIPS) [31]. MIPS functional categories are hierarchical, with top-level functions describing very general protein functions (for example, 'metabolism') and lower levels describing more specific functions (for example, 'proton driven symporter'). The MRF-NB approach was applied to 13 top-level MIPS functional categories and trained on the 3,588 yeast genes that had been assigned at least one of these categories (see Table 1 for details). For the purposes of comparison, we trained *Funckenstein *on the same data sets used for the MRF analysis and compared our cross-validation performance with the cross-validation performance reported previously [26].

**Table 1:** MIPS functional classes used to compare with MRF-NB approach

Functional categories	No. of genes annotated
Cell cycle and DNA processing	600
Cell fate	411
Cell rescue, defense and virulence	264
Cellular transport and transport mechanisms	479
Control of cellular organization	192
Energy	242
Metabolism	1,048
Protein fate (folding, modification, destination)	578
Protein synthesis	335
Regulation of/interaction with cellular environment	193
Transcription	753
Transport facilitation	306
Others (cellular communication/signal transduction mechanism, or protein activity regulation, or protein with binding function or cofactor requirement (structural or catalytic), or transposable elements, viral and plasmid proteins)	81
	
Total	3,588

MIPS, Munich Information Center for Protein Sequences; MRF, Markov random field; NB, naïve bayes.

To train *Funckenstein*'s guilt-by-profiling classifiers, we used protein sequence pattern and complex membership data. For guilt-by-association analysis, we used four types of pairwise biological relationships: pairwise physical and genetic interactions, correlated gene expression (with a correlation coefficient greater than 0.9), and interaction according to a large-scale affinity purification dataset [5]. When using affinity purification-derived interactions for predictions, we considered both the 'spoke' model (interactions defined to exist between bait and prey proteins) and the 'matrix' model (interaction defined not only between bait and prey protein, but also between prey proteins purified with the same bait) [32]. Because the 'matrix' model gave a slightly worse prediction performance (Table 2), we used the 'spoke' model for affinity purification-derived interactions.

**Table 2:** Area under the precision versus recall curve for MIPS function prediction

Individual classifier						*Funckenstein*				MRF-NB
	
		FL				RF + FL				
				
PDT	RF	Spoke model	Spoke model + Pfam	Matrix Model	Matrix model + Pfam	FL spoke model	FL spoke model + Pfam	FL matrix model	FL matrix model + Pfam	
0.504	0.621	0.364	0.451	0.355	0.451	0.651	0.637	0.644	0.635	0.522

FL, functional linkage; MIPS, Munich Information Center for Protein Sequences; MRF, Markov random field; NB, naïve Bayes; PDT, probabilistic decision tree; RF, random forest.

**Figure 1:**
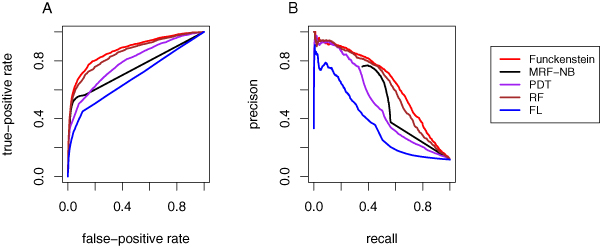
Performance of MIPS function prediction using a previously established benchmark dataset. Results are shown for five different methods: *Funckenstein *(light red); MRF-NB [26] (black); guilt-by-profiling by the RF method alone (dark red); guilt-by-profiling by the PDT method alone (violet); guilt-by-association by the FL method alone (blue). **(a) **True positive rate versus false positive rate at different score thresholds. **(b) **Precision versus recall at prediction score thresholds. MIPS, Munich Information Center for Protein Sequences; MRF, Markov random field; NB, naïve Bayes; PDT, probabilistic decision tree; RF, random forest.

Receiver operating characteristic (ROC) curves reflect the tradeoff between recall and false positive rate that can be achieved by tuning a given method. Here, false positive rate is defined as the fraction of (gene, MIPS category) pairs that were predicted to be positive, among all pairs that are incorrect annotations according to the MIPS annotation. The ROC curves for *Funckenstein *and MRF-NB (Figure 1a) reflect a similar trend to that observed in the precision versus recall curves, with *Funckenstein *yielding an area under the ROC curve (AUC) of 0.88, compared with an AUC of 0.75 for MRF-NB. An AUC of 50% would indicate performance equal to that of random guessing and an AUC of 100% would indicate perfect performance. Although AUC is a measure of overall performance that is used for many applications, it is less appropriate here. Because the total number of unannotated gene/MIPS functional category pairs far exceeds the number of annotations, only performance at extremely low false-positive rates will be relevant to most users. Furthermore, most biologists are more interested in precision (fraction of predictions that are correct) than in false positive rate as defined above. Therefore, we used the precision-recall curve to evaluate prediction performance in all subsequent analyses.

The precision that a given user requires depends on their application. Since each of our predictions is accompanied by a quantitative measure of confidence, users may consider only the highest-precision predictions. Alternatively, users wishing to select candidate genes for further study - for example, for medium-scale genomic experiments - may wish to include a greater number of 'trues' at the expense of precision. Predictions offer a principled form of triage to reduce the search space and reduce the experimental resources required for follow-up study.

Figure 1b, which describes the tradeoff between precision and recall for various score thresholds, shows that *Funckenstein *outperforms MRF-NB in predicting MIPS functional categories. *Funckenstein *yields a higher precision than MRF-NB across the full range of recall values obtained in the MRF-NB study. For example, with a recall of 50% (that is, when 50% of all MIPS annotations are predicted), a precision of 75% is achieved, compared with 67% by MRF-NB. For approximately 44% of the (gene, MIPS category) pairs, MRF-NB did not make a prediction, giving these pairs a score of 0.0. As a result, the precision-recall curve of MRF-NB is truncated at a recall of 56%. With a recall of 55%, *Funckenstein *achieves a precision of 71%, compared with 51% by MRF-NB. Of particular interest to users choosing candidate genes for disease association or for medium-scale screens, *Funckenstein *achieves a higher recall than MRF-NB at every level of precision. As a single overall measure of performance, we computed the area under the precision-recall curve. (In truncated regions of the MRF-NB curve, we modeled recall values as having precision corresponding to that of the nearest observed recall value.) The area under the precision-recall curve of *Funckenstein *was 0.651, greater than the 0.522 observed for the MRF-NB approach.

We also investigated the individual contributions of *Funckenstein *classifiers of each type (guilt-by-profiling or guilt-by-association) in predicting MIPS functional categories. The guilt-by-profiling classifier outperforms the guilt-by-association classifier (Figure 1b) with precision-recall curve areas of 0.621 and 0.364, respectively. In fact, the guilt-by-profiling classifier alone already outperforms MRF-NB (area 0.522). Figure 1 also shows the performance of an alternative PDT guilt-by-profiling method in predicting MIPS functional categories. The RF method is undoubtedly an improvement over the PDT method (area 0.621 versus 0.504). This supports our choice to use the RF method for all other guilt-by-profiling predictions described here.

Combining the two component classifiers led to better performance than was achieved with either alone. However, the improvement of *Funckenstein *over the guilt-by-profiling classifier was modest for the MIPS benchmark (area of 0.651 compared with 0.621 under that of guilt-by-profiling classifier). This could be because of the very limited number of available interactions in this dataset. More substantial improvement would be expected as more relationships are included.

Note that differences in component classifier performance may be due either to algorithmic differences or to the value of the features used for prediction, and the comparison above does not separate these effects. Therefore, we also investigated the effect of transforming gene features into gene-gene relationships for use by the FL classifier. Specifically, we treated shared annotation with a Pfam domain as a gene-gene relationship (information found in expression cluster membership features was already available to the FL classifier as protein interactions). This substantially improved the performance of the FL classifier to an area under the precision-recall curve of 0.451 (versus the previous 0.364 area). However, use of the improved FL guilt-by-association classifier within *Funckenstein *actually reduced its overall performance (area 0.651 versus 0.637). One explanation is that the inclusion of the same evidence type in both component classifiers may lead to 'over-counting' of evidence and, thus, a slightly weaker *Funckenstein*. Thus, it may be important to include component classifiers using relatively independent input features for the success of *Funckenstein*.

### Application to Gene Ontology terms using an expanded integrated dataset

Although it enabled us to compare *Funckenstein *and MRF-NB methods directly, the previously defined set of functional categories has the drawback of being somewhat non-specific. (For example, it is not immediately clear what specific follow-up experiment would test the hypothesis that a gene is involved in 'metabolism'.) Therefore, we sought to apply *Funckenstein *to predict more specific GO functional terms. We also expanded the scope of the data used to make predictions. We assembled data for 21 gene characteristic types and 23 experimentally determined gene-gene relationship types (shown in Tables 3 and 4, respectively).

**Table 3:** Gene attributes used in guilt-by-profiling GO term predictions

Gene characteristic type	No. of characteristics	Source
Phenotype	27	Giaever *et al. *[44]
Phenotype	12	Parsons *et al. *[45]
Phenotype	78	Lum *et al. *[19]
Phenotype	1	Baetz *et al. *[46]
Phenotype	4	Tucker *et al. *[47]
Phenotype	22	Dudley *et al. *[48]
Protein complex	232	Gavin *et al. *[5]
Protein complex	493	Ho *et al. *[6]
Protein complex and cellular localization	8	Ng *et al. *[49]
Protein complex and cellular localization	10	Robert *et al. *[50]
Protein cellular localization	23	Huh *et al. *[21]
Protein cellular localization	26	Kumar *et al. *[38]
Transcriptional regulator	352	Harbison *et al. *[51]
Transcriptional regulator	106	Lee *et al. *[52]
Protein sequence pattern	283	ProDom [53]
Protein sequence pattern	1,930	Pfam [8]
Protein sequence pattern	515	TIGRFAM [54]
Protein sequence pattern	928	PROSITE [11]
Protein sequence pattern	378	PANTHER [55]
Protein sequence pattern	328	PRINTs [10]
Protein sequence pattern	3,092	InterPro [9]
		
Total	8,848	

GO, Gene Ontology.

**Table 4:** Biological relationships used in guilt-by-association GO term predictions

Relationship evidence type	No. of gene pairs with evidence	Source^a^
Affinity capture-MS	19,115	Gavin *et al*. [56]
Two-hybrid	9,598	Ito *et al*. [4]
Synthetic lethality	9,067	Tong *et al*. [57]
Synthetic growth defect	5,131	Pan *et al*. [58]
Biochemical activity	4,822	Ptacek *et al*. [59]
Affinity capture-western	3,584	Kang *et al*. [60]
Epistatic miniarray profile	3,416	Schuldiner *et al*. [61]
Dosage rescue	2,493	Gandhi *et al*. [62]
Synthetic rescue	1,667	Valachovic *et al*. [63]
Phenotypic enhancement	1,468	Myung *et al*. [64]
Reconstituted complex	1,345	Kus *et al*. [65]
Co-purification	898	Stevens *et al*. [66]
Phenotypic suppression	589	Mosch *et al*. [67]
Dosage lethality	373	Branzei *et al*. [68]
Co-fractionation	319	Xu *et al*. [69]
Co-localization	208	Shen *et al*. [70]
Co-crystal structure	72	Cramer *et al*. [71]
Protein-peptide	63	Mariño-Ramirez *et al*. [72]
Affinity capture-RNA	52	Tharun *et al*. [73]
Far western	36	Tsai *et al*. [74]
Dosage growth defect	33	Pan *et al*. [75]
FRET	32	Damelin *et al*. [76]
Protein-RNA	6	Gonsalvez *et al*. [77]
		
Total	55,039	

^a^For each evidence type, a representative reference (the one contributing the most interactions) is shown. GO, Gene Ontology.

Terms in the GO vocabulary are organized within a rooted directed acyclic graph with three branches that describe biological process (BP), molecular function (MF), and cellular component (CC), respectively. Within each GO branch, a child GO term may descend from multiple parental GO terms. The vocabulary is structured such that annotation with a given GO term implies 'propagation' of this annotation to all ancestral terms. We obtained GO annotation for all protein-coding *S*. *cerevisiae *genes from SGD and, for each gene, we assigned ancestral GO terms implied by annotated descendent terms.

For GO term prediction, we selected yeast protein-coding genes annotated by at least one of the attribute types listed in Tables 3 and 4. Out of these 6,368 genes, the numbers of 'verified', 'uncharacterized', and 'dubious' open reading frames were 4,476, 1,312, and 580, respectively, when we initiated the work. In total, 5,790 genes were assigned to at least one GO term, including all 'verified' and 'uncharacterized' open reading frames.

Some GO terms describe extremely general functions; for example, 'catalytic activity' (GO:0003824) had 1,885 associated genes. To focus on specific GO terms with the greatest potential to guide future experimentation, we considered only GO terms assigned to 300 or fewer genes. We excluded GO terms assigned to fewer than three genes, because of the extreme difficulty in developing a classifier with such a limited number of positive training examples. After this filtering, 2,455 GO terms remained. For convenience, we separated GO terms into 12 categories corresponding to all combinations of three GO branches (BP, MF, and CC) and four specificity levels (Table 5). Specificity levels are defined according to the number of annotated genes: 3 to 10, 11 to 30, 31 to 100, and 101 to 300 genes. To avoid circularity due to logical dependency between GO terms, we have not allowed classifiers to predict GO terms using other GO terms. The functional linkage graphs used in guilt-by-association analysis were trained separately for each of the 12 GO categories, and the logistic regression parameter for combining guilt-by-association and guilt-by-profiling predictions was optimized separately for each GO category by maximizing the area under the corresponding precision versus recall curve.

**Table 5:** Distribution of the GO functional terms predicted

GO term	GO term branch			
		
specificity	BP	MF	CC	Total
3 to 10	612	415	223	1,250
11 to 30	355	156	109	620
31 to 100	218	87	87	392
101 to 300	126	23	44	193
Total	1,311	681	463	2,455

BP, biological process; CC, cellular component; GO, Gene Ontology; MF, molecular function.

GO term predictions using both guilt-by-profiling and guilt-by-association classifiers were made for the 6,368 *S*. *cerevisiae *protein-coding genes that are either annotated with a GO term or have at least one gene characteristic or interaction. This resulted in a score for each of 6,368 *S*. *cerevisiae *genes for each of 2,455 GO terms for a total of 15,633,440 quantitative annotation scores.

**Figure 2:**
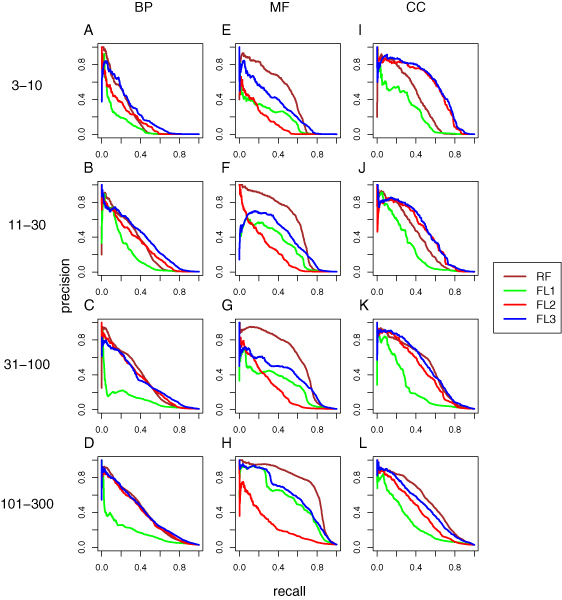
Performance of GO term prediction using either the RF guilt-by-profiling (RF; brown) or FL guilt-by-association classifiers (FL). Three types of FL classifiers were compared: FL1 (green), which used only gene characteristics used in the RF classifier that have been recoded as gene pair characteristics; FL2 (red), which used only 'intrinsic' gene-gene relationships; and FL3 (blue), which used both intrinsic and recoded gene-gene characteristics. **(a-l) **Plots are organized according to GO branch and GO term specificity. FL, functional linkage; GO, Gene Ontology; RF, random forest.

**Figure 3:**
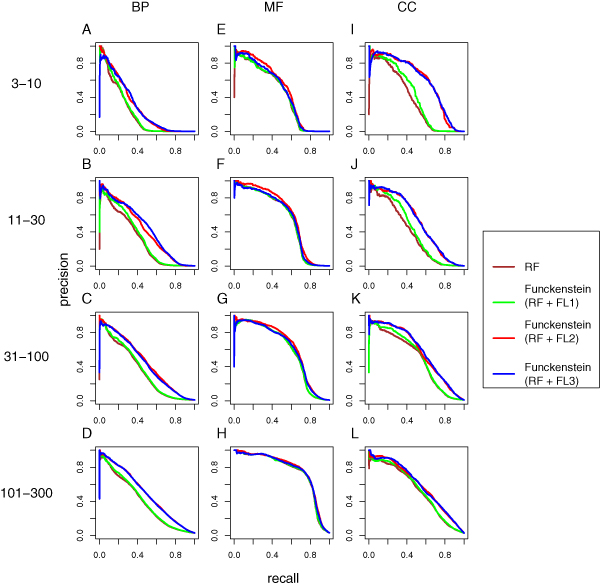
Cross-validation results for *Funckenstein *and the RF guilt-by-profiling component classifier (RF) alone (brown). Three versions of *Funckenstein *were compared, each integrating RF with one of three variants of the FL guilt-by-association classifier (FL): FL1, FL using only relationships derived from shared gene characteristics (green); FL2, FL using only direct gene-gene relationships (red); and FL3, FL using all types of relationship (blue). **(a-l) **Plots are organized according to GO branch and GO term specificity. FL, functional linkage; GO, Gene Ontology; RF, random forest.

We also assessed variants of the FL classifier using relationships defined by shared annotation (as discussed above for MIPS function predictions). Results from the variant FL component classifier were combined with the RF predictions to yield variants of *Funckenstein*. As described above for MIPS function predictions, we evaluated prediction performance by calculating precision and recall at different quantitative score thresholds and by calculating total area under the precision-recall curve within each of the 12 GO categories. Performance was calculated for individual component classifiers, and for variants of *Funckenstein*, the combined classifier (Figures 2 and 3, respectively, with underlying values shown in Table 6).

**Table 6:** The area under the precision versus recall curve for different classifiers

GO branch	GO specificity	RF	FL1*	FL2*	FL3*	Funckenstein (RF + FL1)	Funckenstein (RF + FL2)	Funckenstein (RF + FL3)
BP	3 to 10	0.215	0.111	0.173	*0.245*	0.225	**0.286**	**0.286**
	11 to 30	0.306	0.197	0.296	*0.359*	0.331	0.422	**0.441**
	31 to 100	*0.349*	0.117	0.329	0.33	0.36	**0.454**	0.441
	101 to 300	*0.394*	0.169	0.373	*0.394*	0.405	**0.492**	0.491
MF	3 to 10	*0.44*	0.188	0.134	0.309	0.445	**0.489**	0.463
	11 to 30	*0.557*	0.296	0.216	0.383	0.558	**0.599**	0.565
	31 to 100	*0.596*	0.29	0.232	0.393	0.597	**0.632**	0.61
	101 to 300	*0.75*	0.55	0.24	0.595	0.753	**0.77**	0.762
CC	3 to 10	0.368	0.242	0.546	*0.571*	0.413	**0.597**	0.596
	11 to 30	0.377	0.261	0.441	*0.465*	0.417	**0.523**	0.524
	31 to 100	0.489	0.249	0.44	*0.496*	0.508	0.578	**0.586**
	101 to 300	*0.524*	0.271	0.391	0.468	0.537	0.591	**0.595**

*FL1, FL classifier based on shared gene characteristics in Table 3; FL2, FL classifier based on direct gene-gene relationships in Table 4; FL3, FL classifier based on both relationship types. Entries in bold are the best *Funckenstein *classifier; entries in italics are the best individual classifier. BP, biological process; CC, cellular component; FL, functional linkage; GO, Gene Ontology; MF, molecular function; RF, random forest.

The addition of relationships based on shared annotation improved predictions for MF attributes, but not BP and CC attributes (Figure 2 and Table 6). For example, the area under the precision-recall curve for MF attributes with 101 to 300 genes was 0.55 for the FL guilt-by-association classifier using shared annotation relationships alone, in contrast to 0.24 using only direct gene-gene relationships. By contrast, the FL classifiers for BP and CC attributes with 101 to 300 genes yielded precision-recall areas of 0.169 and 0.271, respectively, using only shared annotation relationships - compared with 0.373 and 0.391, respectively, for the FL classifiers using only direct relationships. It is intuitively reasonable that shared annotation with protein motifs would be most useful for inferring similar molecular function rather than similar BP and CC attributes, and that direct relationships such as protein interaction would be more useful for inferring similar BP and CC annotation. The FL classifier variant that used both shared annotation and direct relationships outperformed variants using either type of relationship alone, especially for predicting MF attributes.

The RF guilt-by-profiling classifier substantially outperformed the best FL guilt-by-association classifier in predicting MF attributes. For example, the precision-recall area of the RF classifier for MF attributes with specificity of 11 to 30 genes was 0.557, in contrast to 0.383 for the best FL classifier, suggesting that the presence of particular sequence motifs (or combinations of sequence motifs) is more helpful in inferring molecular function than are gene-gene relationships. In predicting BP and CC attributes, the opposite was true. The FL classifier outperformed the RF classifier in predicting BP and particularly in predicting CC attributes. For example, for CC attributes with 11 to 30 genes, the precision-recall area of the best FL classifier was 0.465, in contrast to 0.377 by the RF classifier. For BP attributes with 11 to 30 genes, the area for the best FL classifier was 0.359, compared with 0.306 by the RF classifier.

By combining the guilt-by-profiling and guilt-by-association classifiers, *Funckenstein *generally outperforms either classifier alone in all three GO branches (Figure 3 and Table 6), and the combination shows particular synergy for BP and CC attributes. For example, for BP attributes with 31 to 100 genes, the precision-recall area of the best *Funckenstein *classifier is 0.454, compared with 0.349 by the best individual classifier, a 30% improvement. For CC attributes with 31 to 100 genes, the precision-recall area increases by 18% from 0.496 for the best individual classifier to 0.586 by the best *Funckenstein *classifier. The improvement for MF attributes is not substantial, with the best performance coming from the RF classifier, which relies on the presence or absence of protein motif patterns.

Interestingly, the best *Funckenstein *classifier is not based on the best guilt-by-association component classifier. The *Funckenstein *variant based on the FL classifier using only direct relationships excels in most GO categories, and is particularly superior for MF attributes. Consequently, we chose the *Funckenstein *with guilt-by-association classifier using only direct relationships as our final predictor. As discussed above for MIPS function predictions, it appears that complementarity of individual classifiers is more important than use of individually optimized component classifiers.

The final version of *Funckenstein *achieves high prediction precision in predicting GO terms. For example, at a recall rate at 50%, *Funckenstein *achieves a prediction precision for GO terms with 101 to 300 genes of 48%, 89% and 64% for BP, MF and CC branches, respectively. In all three branches, it is clear that these precision levels could greatly facilitate the choice of candidate genes for follow-up experiments - precision levels of only approximately 3% would be expected from unguided 'brute force' experimentation. For the most specific GO terms annotated with only 3 to 10 genes, *Funckenstein *still achieves (at 50% recall) precision rates of 14%, 59%, and 73% for BP, MF, and CC branches, respectively, which may be compared with a corresponding unguided precision of approximately 0.09%.

Because there were no experimentally determined gene-gene relationships for any dubious genes in our data set, performance of the above-mentioned classifiers was based on verified and uncharacterized protein-coding genes (5,790 genes in total). To generate scores for dubious genes, we used scores obtained by the guilt-by-profiling classifier alone. This resulted in a total of 15,569,760 quantitative prediction scores (2,455 GO terms × 6,368 genes).

**Figure 4:**
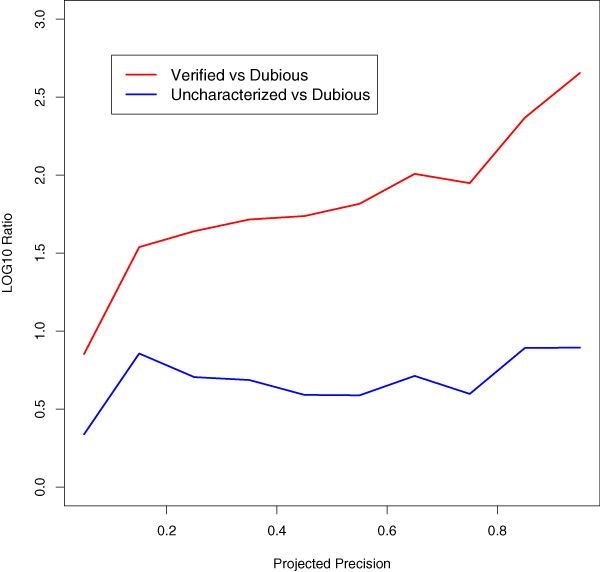
Prediction scores for 'verified', 'uncharacterized', and 'dubious' genes. For 'verified' (red) or 'uncharacterized' (blue) genes, the log ratio of the number of predictions within each score interval (relative to the number for 'dubious' genes) is shown.

To investigate trends associated with verified, uncharacterized, or dubious genes, we counted the number of predictions for genes of each type that fell into each of ten prediction score intervals. Based on the correspondence of prediction score to observed precision, these intervals were chosen to have an expected precision of 0 to 0.1, 0.1 to 0.2, and so on. Within each interval, we compared the number of predictions for verified or uncharacterized genes with that of dubious genes (Figure 4). As expected, uncharacterized and verified genes tended to have higher precision scores than dubious genes, with verified genes tending to have the best scores.

**Figure 5:**
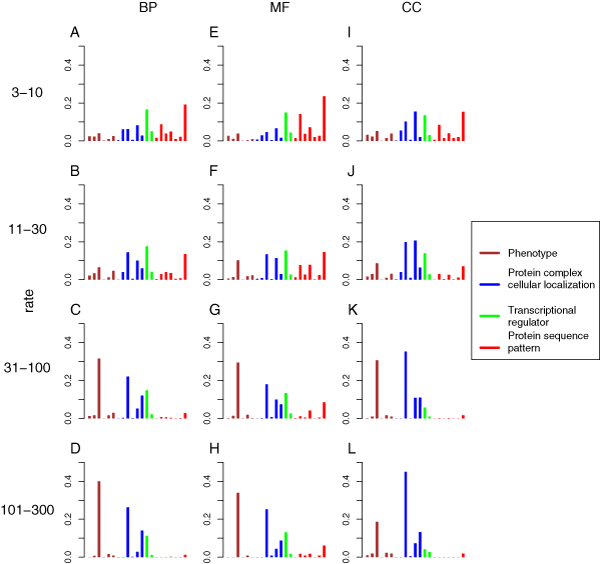
The average frequency of each type of gene characteristic among the five most important variables (see Materials and methods for the variable performance measure): phenotype (brown); protein complex and/or cellular localization (blue); transcription regulation (green); and protein sequence pattern (red). The gene characteristic types are organized according to their order in Table 3. **(a-l) **Plots are organized according to GO branch and GO term specificity. GO, Gene Ontology.

**Figure 6:**
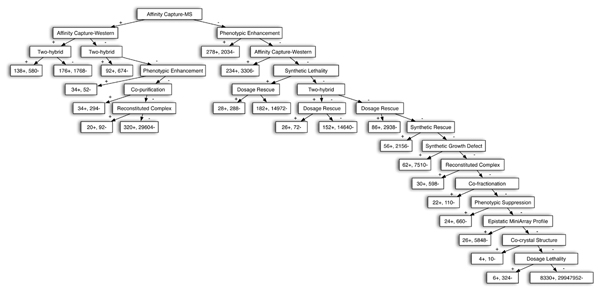
An example of a PDT used to generate a FL graph. This example was trained based on annotations of those BP GO terms that are currently annotated to 3 to 10 genes. BP, biological process; FL, functional linkage; PDT, probabilistic decision tree.

We show in Figure 5 the impact of different evidence types on prediction performance of the guilt-by-profiling classifier. In Figure 6, we show an example decision tree of the guilt-by-association classifier. Detailed discussion of these two figures can be found in the Discussion section.

### Literature-based evaluation of novel GO function predictions

In calculating precision and recall by cross-validation, only predictions corresponding to currently known GO annotations were counted as 'true'. This is conservative in that our current knowledge of gene function in *S*. *cerevisiae *is far from complete, and some predictions considered to be 'false' may actually be true. A high-scoring prediction that is 'false' is most appropriately viewed as a novel prediction. Indeed, novel predictions are the most interesting product of a quantitative gene annotation system.

To assess the best-scoring novel predictions according to current literature, we selected 120 high-scoring novel predictions (the top 10 from each of the 12 GO categories). Within each GO category, we selected predictions in order of score. To avoid over-weighting particular genes or GO terms in our evaluation, we excluded predictions involving a gene or GO term already associated with a higher-scoring prediction within the same category of GO terms. Each novel prediction was assessed by an expert curator within the SGD group (JP, with guidance from JMC).

Each prediction was assigned with one of the following labels: A, 'known correct', that is, having strong supporting evidence in the literature that was not yet captured by a GO annotation in SGD; B, 'likely true', that is, having supporting evidence that is inconclusive; C, 'unclear'; D, 'unlikely to be true', that is, having evidence which either mitigates against the prediction or which suggests an incompatible annotation; or E, 'highly unlikely', that is, having strong evidence that contradicts the association or supports a clearly incompatible annotation (see Table 7 for more detail). We consider predictions labeled 'A' as known correct predictions, and predictions with either 'A' or 'B' as supported.

**Table 7:** Categories for expert classification of novel predictions

Category		Brief description
A	Known true	There is experimental evidence for this annotation, but it is not yet captured by SGD (all supporting literature pre-dates the analysis)
B	Likely true	No experimental evidence exists in the literature, but there is author speculation or sequence similarity support for this annotation
C	Unclear	Experimental evidence is either unavailable or conflicting.
D	Unlikely	The gene product is known to have a related but different function/process and no experimental support was found for the prediction
E	Highly unlikely	Direct experimental evidence against this association, or all other experimental evidence supports an unrelated function/process, or the gene product is part of a well-characterized complex and the predicted component term was to a different well-characterized complex

SGD, *Saccharomyces *Genome Database.

**Figure 7:**
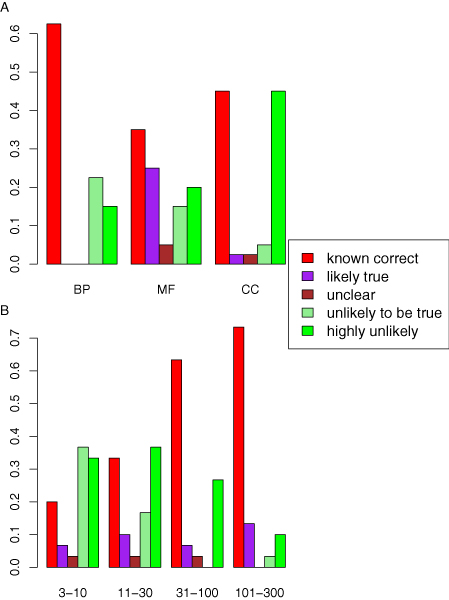
Assessment of 120 novel predictions by an expert curator. Assessments were either 'known correct', 'likely true', 'unclear', 'unlikely to be true' or 'highly unlikely'. **(a) **For each of the three GO branches, the proportion of novel predictions given each assessment. **(b) **For each specificity level, the proportion of novel predictions given each assessment. GO, Gene Ontology.

Results of the assessment, summarized in Figure 7a, show the proportion of novel predictions that are known correct in different GO branches. The fractions of top-scoring predictions that are supported were comparable in each of the three branches - 63%, 60% and 48% for BP, MF and CC GO terms, respectively. The fractions of top-scoring predictions that are known correct were 63%, 35% and 45% for BP, MF and CC attributes, respectively. We note that the top-scoring novel BP predictions were confirmed more frequently than MF and CC predictions (the reverse of performance order observed in cross-validation assessment).

The number of 'known correct' predictions is a conservative estimate of the actual number of correct predictions, since supporting evidence may not be available in the literature. Therefore, the number of predictions that are actually correct is estimated to be between the number of predictions labeled 'A' and the number labeled either 'A', 'B', or 'C'. Thus, our estimates for the true rates of success among top novel predictions were 63%, 35% to 65%, and 45% to 50% for BP, MF and CC GO terms, respectively.

We also examined the success rate of novel predictions as a function of the specificity of the GO terms predicted (Figure 7b). The success rates were 33% to 43% and 20% to 27% for GO terms with specificity 11 to 30 and 3 to 10, respectively. As one might expect, *Funckenstein*'s precision for novel predictions rose substantially for more general GO terms: 73% to 86% and 63% to 71% for GO terms with specificity 101 to 300 and 31 to 100, respectively.

Top novel predictions and their evaluations are listed in Additional data file 1. Only 2 out of these 120 predictions were made for 'uncharacterized' genes, and none were made for dubious genes. Among these 120 predictions, 103 are refinements of original annotations, that is, the target gene has already been annotated with an ancestor GO term of the predicted GO. For example, the correct novel prediction of 'DNA bending activity' (GO:0008301) for *ARG80 *was a refinement, in that *ARG80 *was previously annotated with 'DNA binding' (GO:0003677), the direct parent term of 'DNA bending activity'. The remaining 17 predictions in Additional data file 1 have no annotated ancestor GO terms assigned to the target gene except the root GO term, and are, therefore, particularly interesting novel predictions. For example, *BPT1 *was correctly predicted to have 'ATPase activity, coupled to transmembrane movement of substances' (GO:0042626), and had not previously been annotated with any ancestor of this GO term. Interestingly, out of these 17 particularly interesting novel predictions, 13 were ranked by a SGD expert as 'known true' or 'likely to be true'.

### A resource for browsing predictions and underlying evidence

So that researchers may browse predictions and gain intuition about the evidence underlying specific predictions, we developed an online resource allowing browsing by GO term or gene. This is publicly available at [33].

## Discussion

### Validation of a method combining guilt-by-profiling and guilt-by-association

The *Funckenstein *method yielded substantial improvement over MRF-NB, a previous method combining guilt-by-profiling with guilt-by-association. Indeed, *Funckenstein *achieved a higher prediction precision across all ranges of recall when applied to the same benchmark data (Figure 1b).

Here we generated a more up-to-date collection of integrated data, and applied *Funckenstein *to predict more specific functional terms from the GO vocabulary. The precision versus recall performance of these predictions was assessed by cross-validation and (for top novel predictions) by a follow-up analysis of the literature.

The results suggest that these predictions could be very useful in guiding experimentalists to promising candidate genes. Even the most challenging GO category, highly specific BP terms, yielded a precision of 14% at 50% recall in cross-validation assessment. Importantly, even predictions with 14% (or lower) precision can be extremely useful. To put 14% precision into perspective, if predictions were made at random for a GO term with approximately 6 genes left to be identified, we would expect a precision at 50% recall of approximately 0.1%. Thus, predictions with 14% precision at 50% recall represent a 140-fold increase in the 'hit rate' compared with random testing (which is the rate that would be achieved by an experimentalist using the 'brute force' approach of testing all genes).

In *Funckenstein*, both guilt-by-profiling and guilt-by-association classifiers were based on a decision tree model. Decision-tree based classifiers offer some advantages over other machine learning algorithms. For example, unlike NB [34], decision trees do not assume conditional independence between different data types. Decision trees can also provide intuition for the reasoning behind predictions more readily than support vector machine approaches [35]. In addition, decision trees have good scaling properties in terms of running time and memory usage, which is an important issue when considering thousands of gene characteristics for thousands of genes, and multiple biological relationships among millions of gene pairs.

Much of the success of *Funckenstein *can be attributed to the RF classifier in the guilt-by-profiling classification. The RF classifier is an ensemble of decision trees [27], which has proven to be superior to regular decision trees in many cases, for example, in predicting protein interactions [36]. As we can see from Figure 1, if we had used the simpler PDT rather than the RF classifier, we would not have gained much improvement over MRF-NB. The fact that the guilt-by-association classifier was based on a PDT suggests that further improvements could be obtained using the RF method to obtain the functional linkage graph used in guilt-by-association.

The guilt-by-association classifier was important to *Funckenstein*'s success, especially in predicting BP and CC attributes. While performance of the guilt-by-association component classifier can be improved by transforming gene characteristics into gene-gene relationships, this does not necessarily lead to better performance in the follow-up integration by *Funckenstein*. Indeed, as shown in Figure 3, *Funckenstein *with the FL classifier that used only experimentally determined gene-gene relationships achieved better performance than *Funckenstein *using the version of FL that performed best by itself. Thus, to achieve the best overall performance, it may be important to integrate component classifiers using complementary input features rather than using the input features that optimize performance of each component classifier alone.

### The use of a FL graph significantly improves the performance of guilt-by-association in predicting specific GO functions

In our benchmark study predicting MIPS functions, the guilt-by-profiling classifier played a more dominant role than guilt-by-association. In predicting GO terms, however, the contribution of guilt-by-association was more substantial. This improvement can be attributed both to the increased number of interactions and inclusion of additional relationship types in the GO term prediction effort, and to the fact that the GO terms were generally more specific. When the benchmark data set for MRF-NB was established, the total number of relationships, including gene expression correlation and co-membership within a protein complex, was only 9,976. By contrast, our GO predictions used 55,039 interactions reported by BioGRID [37]. The number of relationship types also increases from 4 when predicting MIPS functions to 23 when predicting GO terms.

We also found that the relative contribution of guilt-by-association in GO term predictions increases as GO terms become more specific. This coincides with the decreased performance of the guilt-by-profiling RF classifier for more specific GO terms, for which fewer positive training examples (that is, genes previously annotated with the GO term) are available. By contrast, guilt-by-association has the potential to be successful given only a single positive training example gene with a given GO term, since the 'transfer rules' are learned from the many GO terms within each GO category.

The guilt-by-association classifier performs well for both CC and BP GO terms, and even outperforms the RF classifiers in some of the CC and BP subcategories; however, its performance is much poorer in the MF categories than that of the RF classifier. This may be explained by the fact that CC and BP GO terms more often have corresponding relationships with other genes in the genome, while the MF GO terms focus on describing the catalytic activity or structural role of an individual protein.

### Assessing the value of different evidence types in predicting function

The RF classifier is not only useful in prediction, but can also be used to assess the value of particular variables in inferring a given function [27] (see Materials and methods). By ranking gene characteristics according to their importance score, we gain intuition about the gene characteristics that were most useful for inferring a particular function.

For each GO term, we identified the five variables that were most useful in the corresponding RF classifier (see Materials and methods for details). Within each of the 12 GO categories, we then calculated the frequency with which each gene characteristic type was observed among the set of most useful variables (Figure 5). For GO terms assigned to 31 or more genes, we find the protein subcellular localization study by Huh and coworkers [21] and a phenotyping study by Lum and colleagues [19] to be the most useful sources of gene characteristics. In addition, the Huh *et al*. study was the most useful type of gene characteristic in predicting CC GO terms, while the Lum *et al*. study was the most useful gene characteristic type in prediction of BP and MF GO terms. It is intuitively reasonable that the Huh *et al*. study of subcellular localization should be useful in predicting CC GO terms, since CC GO terms often correspond directly to subcellular localizations measured within Huh *et al*.

It is easy to rationalize the value of the Lum *et al*. phenotyping study in predicting BP GO terms, since genes within the same biological pathway or process are known to often exhibit similar phenotypes. However, it was surprising to find that a phenotyping study was dominant in predicting general molecular functions. Phenotypes in Lum *et al*. often correspond to the many genes, so these phenotypes may be useful in combination with other terms, even if the Lum phenotypes alone are not sufficient to predict the target GO term accurately.

Some gene characteristics may also be negative predictors of molecular function. For example, 124 *S*. *cerevisiae *genes are annotated with 'RNA polymerase II transcription factor activity' (GO:0003702). Among 1,453 genes with the gene characteristic 'Huh:nucleus', 106 are annotated with the target GO term; by contrast, among the remaining genes without this characteristic, only 18 are annotated with the target GO term, so that not having 'Huh:nucleus' is particularly valuable as a 'negative predictor' of GO:0003702.

For predicting specific GO terms assigned to 30 or fewer genes, we find no gene characteristic type dominates in terms of variable importance; almost all gene characteristic types are represented among the most useful variables of at least one GO category. Yet there is a tendency for protein sequence patterns to become more useful as GO terms become more specific, within each of the three GO branches. For example, the MF term 'aldehyde reductase activity' (GO:0004032) is annotated to only four *S*. *cerevisiae *genes, each sharing a Prosite sequence motif 'PS00063' that is unique to these four genes, making it the best predictor of this function. In another example, the BF term 'negative regulation of microtubule polymerization or depolymerization' (GO:0031111) is assigned to eight genes, six with a conserved DH domain (Interpro 'IPR000219') involved in Rho GTPase interaction and activation that has been assigned to only seven *S*. *cerevisiae *genes. A third example is the CC term 'proteasome core complex, alpha-subunit complex (sensu Eukaryota)' (GO:0019773) that has been assigned to seven *S*. *cerevisiae *genes. Each shares a protease alpha unit domain, Interpro 'IPR000426', that has been assigned only to these same seven *S*. *cerevisiae *genes. Although protein sequence information is valuable in all GO branches and is increasingly useful for more specific GO terms, it is far from dominant in terms of variable importance. Thus, it is necessary to integrate multiple sources of experimental evidence.

The prediction importance of gene characteristics within the same class (each indicated by color in Figure 5) can be very different. For example, data from Lum *et al*., Huh *et al*., Roberts *et al*., and Interpro are, respectively, the best characteristic types within each of the five characteristic classes. Within the same characteristic class, we can assess value of variables in predicting gene function as one surrogate measure of the corresponding quality of each experiment. It must be noted that this analysis does not reveal the reasons for improved value in predicting function, which may result from differences in sensitivity, specificity or coverage of the corresponding experiments. Furthermore, a data set that is least useful for predicting function may be ideal for other purposes. With these caveats, we note one example from the comparison of characteristic types: among protein subcellular localization studies, gene characteristics derived from the study by Huh and coworkers study were substantially more useful than those of Kumar and colleagues [38] in all 12 GO categories (Figure 5).

It is less clear how one should establish the importance of particular variables in generating the FL graph used in guilt-by-association. However, we can still obtain some intuition by examining the PDT classifier used to generate the FL graph. For example, in the PDT classifier developed for the most specific BP GO category, we can see (Figure 6) that a protein pair with 'Affinity capture-MS', 'Affinity capture-western', and 'two-hybrid' evidence is more likely to share a specific BP function than another pair with 'Affinity capture-MS' and only one of 'Affinity capture-western' or 'two-hybrid' evidence. The corresponding number of functionally linked ('+') and non-functionally linked ('-') gene pairs within the training set were (138+, 580-) for pairs with all three supporting evidence types. The decision tree structure is slightly different in different GO categories, with more general GO categories having more complex structures. However, the 'Affinity capture-MS' tends to be a top choice within decision trees used to generate the FL graph.

### Directions for further improvement

Novel predictions made by *Funckenstein *were sufficiently accurate to be useful in guiding experimental research according to current literature. However, we would like to understand why some top *Funckenstein *predictions were ranked as 'highly unlikely' by the SGD expert. Because highly specific terms are expected to be most useful to experimentalists, we focused on predictions for the most specific GO categories (assigned to 3 to 10 genes). Among the 30 such novel predictions of the most specific GO categories, 10 were considered 'highly unlikely' in light of current literature. These ten fell within each of the GO branches (three, five, and two predictions corresponding to MF, CC, and BP branches, respectively; see Additional data file 1). According to the contribution of the RF and guilt-by-association classifier to the final prediction scores of these ten predictions, we can divide them into two classes: predictions with low RF but high guilt-by-association scores (corresponding to the genes *VPH1*, *SUI2*, *SPT7*, and *TIF5*); and predictions with high RF but low guilt-by-association scores (corresponding to the genes *SFH1*, *MSH3*, *TPS2*, *LSM1*, *MSH6*, and *MLH2*). Predictions in the former class all corresponded to CC GO terms. In each of these four cases, genes were known to encode members of a protein complex, and membership in a different subunit of the same complex was predicted (Additional data file 1). For example, *VPH1 *was predicted to have function 'hydrogen-transporting ATPase V1 domain' (GO:0000221), while according to SGD it is a 'subunit of vacuolar-ATPase V0 domain' (a different subunit of the same complex). Thus, it is no surprise that *VPH1 *was found in multiple high-throughput experiments to interact with all eight *S*. *cerevisiae *genes in the V1 subunit, leading to a high guilt-by-association score and a mistaken prediction. This mistake might have been avoided, given the knowledge that *VPH1 *is annotated to the ATPase V0 domain. However, we had avoided using GO terms to predict other GO terms, to avoid complications arising from strong dependencies between GO terms [23]. This example suggests, however, that we might gain by using GO terms that are strongly anti-correlated with the target GO term as a predictor.

We also examined six top-scoring predictions with high RF but low guilt-by-association scores. All six can be attributed to conserved protein domains. For example, *SFH1 *was predicted to have 'phosphatidylinositol transporter activity' (GO:0008526), while SGD annotation describes *SFH1 *as a 'putative homolog of Sec14p, which is a phosphatidylinositol/phosphatidylcholine transfer protein involved in lipid metabolism'. All five *S*. *cerevisiae *genes currently annotated with 'phosphatidylinositol transporter activity' share the Pfam patterns 'PF00650 (CRAL/TRIO domain)' and 'PF03765 (CRAL/TRIO N-term domain)', as well as the Prosite pattern 'PS50191 (CRAL-TRIO lipid binding domain profile)'. Because *SFH1 *is the only *S*. *cerevisiae *gene other than these five genes to have these domains and sequence patterns, *Funckenstein *assigned the target function with very high confidence. However, a previous study was unable to record any detectable phosphatidylinositol/phosphatidylcholine transfer activity for Sfh1p in cytosolic extracts, even when recombinant Sfh1p was added as a supplement at micromolar concentrations [39], although they could not exclude the possibility that the activity might exist under some other condition. Assuming that this was a mis-prediction, it is not clear how such a mis-prediction might be avoided in future.

## Conclusion

We have developed a combined algorithm named '*Funckenstein' *that combines both gene characteristics and gene-gene relationships to predict functions. *Funckenstein *shows substantial improvement over a promising previous method in predicting MIPS functions of *S*. *cerevisiae *genes. We have therefore applied *Funckenstein *to systematically predict 2,455 more specific GO terms for *S*. *cerevisiae *genes. In cross-validation *Funckenstein *achieves high precision within all three GO branches, even for the most specific GO terms. *Funckenstein *has generated a large number of novel predictions that can be readily explored by experimentalists. The assessment of the top 120 novel predictions by an SGD expert suggests that the resulting novel predictions have the potential to be extremely useful to experimentalists.

## Materials and methods

### Prediction methods

#### The probabilistic decision tree classifier

The PDT is a simple and effective tool for modeling the probability of one variable describing an object conditioned on other known characteristics of that object [40]. The decision tree is a graph describing the recursive division of a set of objects into successively smaller partitions. For a detailed description, see [28,40]. In short, all objects in the training set are initially assigned to the root node. Objects are recursively split into smaller partitions on the basis of the value of a particular predictive variable. The predictive variable used to split objects at each node was chosen based on the Gini impurity measure [28], a measure of uniformity of objects with respect to a given variable of interest. To avoid overfitting, we implemented an 'early stopping' criterion to decide whether the next proposed split provides sufficient information to justify the accompanying increase in model complexity. For the predictive variable offering the largest reduction in Gini impurity, we compute the corresponding hypergeometric distribution probability:

P=NT!⋅NF!⋅NL!⋅NR!N!⋅NTL!⋅NTR!⋅N!FL⋅NFR!

where *N *is the total number of genes in the parent node, and *N*_*T *_and *N*_*F *_are the total number of genes in the parent node annotated with and without the target GO term, respectively, while *N*_*TL *_and *N*_*FL*_, *N*_*TR *_and *N*_*FR *_are the corresponding numbers at the left and right daughter node given the split, respectively. We reject the split and determine the node to be a 'leaf node' if the hypergeometric distribution probability *P *> α (with α = 0.01 except where otherwise specified). We compute the probability of a given gene *i *to be annotated with the target GO term *j *as:

$$P(i,j)=\frac{N_{T}+P_{T}\cdot \Psi }{N_{T}+N_{F}+\Psi }$$

where *N*_*T *_and *N*_*F *_are as defined above, *P*_*T *_is the overall fraction of genes annotated with the target GO term and Ψ is the number of pseudocounts used to correct for small sample sizes (Ψ = 1 for all results described).

#### Random forest classifier

Whereas the PDT classifier produces only one tree as a predictive model for a given GO term, a RF classifier is composed of an ensemble of many decision trees. Each decision tree in the 'forest' differs from the PDT in that: training genes at the root node are selected by bootstrap resampling (sampling with replacement) from the full training set; at every node, only a fraction of available object characteristics are assessed as candidates for splitting the next node; and there is no early-stopping criterion (since the process of averaging over diverse trees is an intrinsic safeguard against overfitting [27]). The score for a given candidate gene and GO term is then the fraction of trees trained on that GO term that classify that gene as positive. More details on the RF classifier can be found in [27].

Because the extremely large number of predictive variables available in our data set (8.848 for the GO term prediction study) impacts the memory and time efficiency of the RF method, we introduced modifications to the RF method. We performed feature selection to eliminate variables that were marginally uninformative. For feature selection we examined the set of unique genes at the root node and computed the hypergeometric distribution probability *P *(as defined in the PDT description above), retaining only those characteristics with *P *< α. Because of the bootstrap resampling in selecting training genes, the selected features may vary between trees. We also imposed the same early stopping criterion and method for calculating a probability score at each leaf node that were described for PDTs above. The final RF score for a given gene and GO term was then the average of the probability scores at leaf nodes corresponding to that gene across all trees in the forest trained on that GO term.

There are three free parameters of the RF classifier: the number of random variables at each split, the threshold for feature selection, and the threshold for early stopping. Optimization of these parameters is described below in the 'Training and cross-validation' section.

We also estimated the importance of each variable in predicting each given GO term. Variable importance was computed using Gini impurity (also used above in the context of PDTs). Specifically, importance of a given variable is the sum of the reduction in Gini impurity over all appearances of that variable over all decision trees in the forest [27].

#### Guilt-by-association classifier

Guilt-by-association was performed using a FL graph, in which each edge in the graph is assigned a weight related to the probability that two genes share a common GO term whose specificity (number of genes) is within the specificity range of a given GO category [29]. The FL graph is then used to assign scores to particular gene and GO term combinations. We produced a separate FL graph for each of 12 GO categories, corresponding to each combination of three GO branches (BP, MF, and CC) and four specificity levels (3 to 10, 11 to 30, 31 to 100, and 101 to 300). We used the PDT method to produce each FL graph. Unlike PDTs predicting specific GO terms where the objects under study are genes, here the objects of interest are gene pairs. The variable to be predicted for each gene pair is the answer to the question 'are these two proteins functionally linked, that is, do they share any one of the specific GO terms in a specified GO category?' The attributes used for this classifier are gene-gene relationships, such as protein or genetic interactions. To limit over-fitting, we used the same PDT early-stopping criterion described in the context of function prediction, with threshold 10^-8^. Thus, for each GO category, each pair of *S*. *cerevisiae *genes is assigned a FL 'weight' reflecting the probability that the gene pair shares a specific GO term in that category. To score the annotation of a particular candidate gene with a particular GO term, we retrieve FL weight scores for all pairings of the candidate gene with genes known to have the GO term and average the top three FL scores.

#### Combining guilt-by-profiling and guilt-by-association scores

Guilt-by-profiling and guilt-by-association classifier scores are combined by a simple logistic regression model with one free parameter. For a given gene *i *and a target function *j*, let the probability score computed by the guilt-by-profiling and guilt-by-association classifiers be *P*_*GBP*_(*i*, *j*) and *P*_*GBA*_(*i*, *j*), respectively, then the corresponding combined score *P*(*i*, *j*) is defined as:

$$\log(\frac{P(i,j)}{1-P(i,j)})=w\cdot \log(\frac{P_{GBP}(i,j)}{1-P_{GBP}(i,j)})+(1-w)\cdot \log(\frac{P_{GBA}(i,j)}{1-P_{GBA}(i,j)})$$

where *w *is optimized over the range from 0 to 1 based on area under the precision versus recall curve obtained in cross-validation.

#### Training and cross-validation

For both PDT and RF classifiers, we performed ten-fold cross-validation to obtain a probability score for each gene and used these scores to assess performance. For the RF classifier, we obtained an 'out-of-bag' score for every gene. Each tree in the forest uses only approximately 66% of genes in training. Therefore, for each gene we identified the approximately 33% of trees that did not use that gene in training and averaged the scores for that gene to obtain its 'out-of-bag' score.

We optimized the free parameters of the RF classifier according to the performance of 'out-of-bag' scores. To evaluate performance of a set of scores, we obtain at multiple score thresholds the number of true positive (*TP*), false positive (*FP*), true negative (*TN*), and false negative (*FN*) genes at the given threshold. *TP *(*FP*) is defined as the number of proteins with a score greater than the threshold, which are (are not) currently annotated with target function. *FN *(*TN*) is defined as the number of proteins with a score below the threshold that are (are not) currently annotated with the target function. Thus, we can compute 'precision':

$$P_{precision}=\frac{TP}{TP+FP}$$

'recall' or 'true positive rate':

$$P_{recall}=P_{true}=\frac{TP}{TP+FN}$$

and 'false positive rate':

$$P_{false}=\frac{FP}{FP+TN}$$

Thus, to assess the performance of a given set of scores, we can plot *P*_*precision *_versus *P*_*recall*_, or *P*_*true *_versus *P*_*false *_(the latter is often called a ROC curve). Although the ROC curve is widely used in evaluating the performance of a classifier, it is not the best measure for this application. Because annotations are sparse, only very low false positive rates correspond to predictions with a level of precision that is likely to be useful. Therefore, we used the area under the precision-recall curve to optimize the three free parameters of the RF classifier and the single free parameter of the logistic regression procedure used to combine guilt-by-profiling and guilt-by-association scores. We also used the precision-recall curve to compute the 'projected precision' corresponding to a given prediction score. First, we computed its corresponding recall. Then, we identified the maximal precision at or greater than the recall in the precision-recall curve, and used that precision as the 'projected precision' corresponding to a given prediction score.

### Predictions of MIPS functions using a previously defined benchmark data set

#### Data sources

For comparison with the earlier MRF-NB method, we used the benchmark data set to which it was previously applied. This used the MIPS catalog of functional categories ('FunCat') [41]. Although this catalog currently includes 28 top-level functional categories that describe general functions, we used the 13 functional classes derived previously from MIPS [26] (see Table 1 for detailed description). We also predicted only for the 3,588 *S*. *cerevisiae *genes annotated with at least one of the 13 function classes, as was done previously. We used the same information used in training the MRF-NB model, including Pfam domain and tandem affinity purification (TAP) complexes [5], protein and genetic interactions derived from MIPS, and gene expression correlation. We downloaded this information from the website corresponding to the previous MRF-NB publication without any post-processing [42].

We trained PDT and RF guilt-by-profiling classifiers using gene characteristics, that is, protein domain and complex information. The FL graph for the guilt-by-association classifier was trained using protein and genetic interactions from MIPS, gene expression correlation, and TAP complex interaction based on the 'spoke' model [32].

### GO function prediction

#### Sources of data

We downloaded associations of GO terms with *S*. *cerevisiae *genes from SGD [43] in December 2006. We also downloaded the GO directed acyclic graph structure from the GO database (November 30, 2006 release) and used it to 'propagate' associations so association to a particular GO term was propagated to all ancestral GO terms. We identified 2,530 GO terms that are associated with at least three *S*. *cerevisiae *genes according to the propagated annotation. We defined a measure of functional specificity for each GO term by counting the number of genes currently associated with that GO term. Then, following the same strategy that was used in a recent critical assessment of *Mus musculus *gene functions (unpublished data), we divided these GO terms into 12 subcategories according to GO branches and GO specificities (3 to 10, 11 to 30, 31 to 100, and 101 to 300 genes). The total number of GO terms in these 12 categories was 2,455. The parameters of the RF classifier and *Funckenstein *were trained independently for each of the 12 GO subsets.

For guilt-by-profiling predictions, we collected a large number of gene attributes derived both from sequence analysis and high-throughput experimental data (see Table 3 for details). For guilt-by-association predictions, we downloaded experimentally determined interaction types from the BioGRID database [37]. This database defines 23 protein and genetic interaction types, for example, yeast two-hybrid protein interaction and synthetic lethal genetic interaction (see Table 4 for details), corresponding to a total of 55,039 unique gene pairs with some relationship. We also computed shared annotation relationships for all gene characteristics in Table 3. Within a given class of gene characteristics, a gene pair was considered to have shared annotation if the number of shared characteristics was more than 90% of the number of unique characteristics held by either protein.

## Abbreviations

AUC, area under the ROC curve; BP, biological process; CC, cellular component; FL, functional linkage; GO, Gene Ontology; MF, molecular function; MIPS, Munich Information Center for Protein Sequences; MRF, Markov random field; NB, naïve Bayes; PDT, probabilistic decision tree; ROC, Receiver operating characteristic; RF, random forest; SGD, *Saccharomyces *Genome Database.

## Competing interests

The authors declare that they have no competing interests.

## Authors' contributions

WT, LVZ, MT, and FPR conceived the study. WT, MT, LVZ, ODK, and FR developed the methodology for prediction and evaluation. WT, LVZ, and ZW assembled the data. WT performed the analysis. MT and LVZ carried out early variations of the analysis. WT and FDG constructed the website. JP performed analysis of novel predictions with guidance from JMC. WT and FR drafted the manuscript. All authors read and approved the final manuscript.

## Additional data files

The following additional data are available with the online version of this paper. Additional data file 1 is a table listing the results of expert evaluation of GO predictions by *Funckenstein*.

## Supplementary Material

Additional data file 1 Expert evaluation of GO predictions by *Funckenstein*. Click here for file
