# Supplementary material for: Combining guilt-by-association and guilt-by-profiling to predict Saccharomyces cerevisiae gene function
Source: Genome Biol. 2008 Jun 27;9(Suppl 1):S7. doi: 10.1186/gb-2008-9-s1-s7 (PMC2447541; doi:10.1186/gb-2008-9-s1-s7)
Supplement: Additional data file 1 — Expert evaluation of GO predictions by Funckenstein. [file gb-2008-9-s1-s7-S1.pdf]

Supplementary Table 1. Expert evaluation of GO predictions by *Funckenstein*.

Gene Name	Gene feature type	Predicted GO terms	Annotated ancestor GO terms	Expert evaluation
**MF 3-10 genes**				
YKL091C	Verified	GO:0008526 "phosphatidylinositol transporter activity"	NO	highly unlikely
MSH3	Verified	GO:0032134 "mispaired DNA binding"	YES	highly unlikely
TPS2	Verified	GO:0003825 "alpha,alpha-trehalose-phosphate synthase (UDP-forming) activity"	YES	highly unlikely
GYL1	Verified	GO:0005097 "Rab GTPase activator activity"	NO	likely true
SHS1	Verified	GO:0005545 "phosphatidylinositol binding"	YES	likely true
PUS9	Verified	GO:0004730 "pseudouridylate synthase activity"	YES	unlikely to be true
PAC2	Verified	GO:0008017 "microtubule binding"	YES	unlikely to be true
CSE1	Verified	GO:0008320 "protein carrier activity"	YES	known true
ALD4	Verified	GO:0004028 "3-chloroallyl aldehyde dehydrogenase activity"	YES	unlikely to be true
ITC1	Verified	GO:0003709 "RNA polymerase III transcription factor activity"	NO	unlikely to be true
**MF 11-30 genes**				
YKL091C	Verified	GO:0005319 "lipid transporter activity"	NO	unclear
UBC9	Verified	GO:0004840 "ubiquitin conjugating enzyme activity"	YES	highly unlikely
TPS2	Verified	GO:0046527 "glucosyltransferase activity"	YES	highly unlikely
SHS1	Verified	GO:0035091 "phosphoinositide binding"	YES	likely true
SLA2	Verified	GO:0030276 "clathrin binding"	YES	known true
MSH3	Verified	GO:0030554 "adenyl nucleotide binding"	YES	likely true
PUS9	Verified	GO:0016836 "hydro-lyase activity"	YES	unlikely to be true
GYL1	Verified	GO:0005099 "Ras GTPase activator activity"	NO	likely true
RPO21	Verified	GO:0016455 "RNA polymerase II transcription mediator activity"	YES	unlikely to be true
ARG80	Verified	GO:0008301 "DNA bending activity"	YES	known true
**MF 31-100 genes**				
SSY1	Verified	GO:0005275 "amine transporter activity"	YES	highly unlikely
GYL1	Verified	GO:0030695 "GTPase regulator activity"	NO	likely true
BPT1	Verified	GO:0042626 "ATPase activity, coupled to transmembrane movement of substances"	NO	known true
LSM1	Verified	GO:0031202 "RNA splicing factor activity, transesterification mechanism"	YES	highly unlikely
SSE1	Verified	GO:0051082 "unfolded protein binding"	NO	known true
NUT1	Verified	GO:0016251 "general RNA polymerase II transcription factor activity"	NO	known true
SHS1	Verified	GO:0005543 "phospholipid binding"	YES	likely true
YIR042C	Uncharacterized	GO:0008415 "acyltransferase activity"	NO	unclear
TPS2	Verified	GO:0016757 "transferase activity, transferring glycosyl groups"	YES	highly unlikely
DOC1	Verified	GO:0016879 "ligase activity, forming carbon-nitrogen bonds"	YES	known true
**MF 101-300 genes**				

KIC1	Verified	GO:0016773 "phosphotransferase activity, alcohol group as acceptor"	YES	known true
IKS1	Uncharacterized	GO:0016772 "transferase activity, transferring phosphorus-containing groups"	NO	likely true
YEF3	Verified	GO:0016887 "ATPase activity"	NO	known true
NPR1	Verified	GO:0004672 "protein kinase activity"	YES	known true
BPT1	Verified	GO:0016462 "pyrophosphatase activity"	NO	known true
GYL1	Verified	GO:0030234 "enzyme regulator activity"	NO	likely true
YME1	Verified	GO:0016817 "hydrolase activity, acting on acid anhydrides"	YES	likely true
SSE1	Verified	GO:0017111 "nucleoside-triphosphatase activity"	NO	known true
CTF18	Verified	GO:0016818 "hydrolase activity, acting on acid anhydrides, in phosphorus-containing anhydrides"	NO	known true
CLF1	Verified	GO:0003723 "RNA binding"	YES	known true
**CC 3-10 genes**				
VPH1	Verified	GO:0000221 "hydrogen-transporting ATPase V1 domain"	YES	highly unlikely
SUI2	Verified	GO:0005851 "eukaryotic translation initiation factor 2B complex"	YES	highly unlikely
SPT7	Verified	GO:0005671 "Ada2/Gcn5/Ada3 transcription activator complex"	YES	highly unlikely
SHS1	Verified	GO:0000399 "bud neck septin structure"	YES	known true
TIF5	Verified	GO:0005852 "eukaryotic translation initiation factor 3 complex"	YES	highly unlikely
RPG1	Verified	GO:0043614 "multi-eIF complex"	YES	known true
VPS8	Verified	GO:0030897 "HOPS complex"	YES	unlikely to be true
LSM1	Verified	GO:0005688 "snRNP U6"	YES	highly unlikely
RPN10	Verified	GO:0008541 "proteasome regulatory particle, lid subcomplex (sensu Eukaryota)"	YES	unclear
CHL4	Verified	GO:0000817 "COMA complex"	YES	unlikely to be true
**CC 11-30 genes**				
RPB3	Verified	GO:0005736 "DNA-directed RNA polymerase I complex"	YES	highly unlikely
SHS1	Verified	GO:0032153 "cell division site"	YES	known true
TAF1	Verified	GO:0000124 "SAGA complex"	YES	highly unlikely
LSM4	Verified	GO:0005682 "snRNP U5"	YES	highly unlikely
RPO21	Verified	GO:0000119 "mediator complex"	YES	highly unlikely
PRE1	Verified	GO:0005838 "proteasome regulatory particle (sensu Eukaryota)"	YES	highly unlikely
SPT3	Verified	GO:0005669 "transcription factor TFIID complex"	YES	highly unlikely
SPT8	Verified	GO:0046695 "SLIK (SAGA-like) complex"	YES	highly unlikely
LSM1	Verified	GO:0046540 "U4/U6 x U5 tri-snRNP complex"	YES	highly unlikely
PRP19	Verified	GO:0005685 "snRNP U1"	YES	highly unlikely
**CC 31-100 genes**				
PRP19	Verified	GO:0030532 "small nuclear ribonucleoprotein complex"	YES	highly unlikely
SRP1	Verified	GO:0031965 "nuclear membrane"	YES	known true
NGG1	Verified	GO:0016591 "DNA-directed RNA polymerase II, holoenzyme"	YES	highly unlikely
CLF1	Verified	GO:0005681 "spliceosome complex"	YES	known true
TAF1	Verified	GO:0000123 "histone acetyltransferase complex"	YES	known true
ABP1	Verified	GO:0015629 "actin cytoskeleton"	YES	known true
VMA10	Verified	GO:0000329 "vacuolar membrane (sensu Fungi)"	YES	known true

LSM1	Verified	GO:0005684 "major (U2-dependent) spliceosome"	YES	highly unlikely
TID3	Verified	GO:0005819 "spindle"	YES	known true
SPC25	Verified	GO:0000922 "spindle pole"	YES	known true
**CC 101-300 genes**				
SRP1	Verified	GO:0005635 "nuclear envelope"	YES	known true
ABP1	Verified	GO:0044430 "cytoskeletal part"	YES	known true
VMA10	Verified	GO:0000322 "storage vacuole"	YES	known true
TID3	Verified	GO:0005856 "cytoskeleton"	YES	known true
SHS1	Verified	GO:0030427 "site of polarized growth"	YES	known true
VMA2	Verified	GO:0000324 "vacuole (sensu Fungi)"	YES	known true
SPC25	Verified	GO:0015630 "microtubule cytoskeleton"	YES	known true
SEC18	Verified	GO:0005794 "Golgi apparatus"	YES	known true
ERP1	Verified	GO:0016021 "integral to membrane"	YES	likely true
RPO21	Verified	GO:0005667 "transcription factor complex"	YES	highly unlikely
**BP 3-10 genes**				
MSH6	Verified	GO:0000735 "removal of nonhomologous ends"	YES	highly unlikely
CFT2	Verified	GO:0030847 "transcription termination from Pol II promoter, RNA polymerase(A)-independent"	YES	unlikely to be true
RAD51	Verified	GO:0000730 "DNA recombinase assembly"	YES	unlikely to be true
GAL3	Verified	GO:0019388 "galactose catabolism"	YES	unlikely to be true
CDC2	Verified	GO:0000731 "DNA synthesis during DNA repair"	YES	known true
GLK1	Verified	GO:0006000 "fructose metabolism"	YES	unlikely to be true
YSH1	Verified	GO:0030846 "transcription termination from Pol II promoter, RNA polymerase(A) coupled"	YES	unlikely to be true
GAL83	Verified	GO:0042149 "cellular response to glucose starvation"	YES	known true
MLH2	Verified	GO:0000710 "meiotic mismatch repair"	YES	highly unlikely
DMC1	Verified	GO:0030491 "heteroduplex formation"	YES	known true
**BP 11-30 genes**				
PEP12	Verified	GO:0006906 "vesicle fusion"	YES	known true
EXO84	Verified	GO:0048278 "vesicle docking"	YES	known true
SHS1	Verified	GO:0007120 "axial bud site selection"	YES	known true
HDA1	Verified	GO:0016575 "histone deacetylation"	YES	known true
STE20	Verified	GO:0007266 "Rho protein signal transduction"	YES	known true
RAD6	Verified	GO:0000209 "protein polyubiquitination"	YES	known true
BOS1	Verified	GO:0006891 "intra-Golgi vesicle-mediated transport"	YES	unlikely to be true
CFT2	Verified	GO:0006369 "transcription termination from RNA polymerase II promoter"	YES	unlikely to be true
GLC7	Verified	GO:0031123 "RNA 3'-end processing"	YES	known true
**BP 31-100 genes**				
PEP12	Verified	GO:0006944 "membrane fusion"	YES	known true
QRI8	Verified	GO:0032446 "protein modification by small protein conjugation"	YES	known true
LSM1	Verified	GO:0000398 "nuclear mRNA splicing, via spliceosome"	YES	highly unlikely

SSY1	Verified	GO:0015837 "amine transport"	YES	known true
RGD1	Verified	GO:0007264 "small GTPase mediated signal transduction"	YES	known true
SHS1	Verified	GO:0000282 "bud site selection"	YES	known true
KSS1	Verified	GO:0006468 "protein amino acid phosphorylation"	YES	known true
DOC1	Verified	GO:0006512 "ubiquitin cycle"	YES	known true
SPC97	Verified	GO:0007051 "spindle organization and biogenesis"	YES	known true
CRM1	Verified	GO:0051170 "nuclear import"	YES	highly unlikely
**BP 101-300 genes**				
CDC3	Verified	GO:0051704 "interaction between organisms"	NO	known true
LSM1	Verified	GO:0008380 "RNA splicing"	YES	highly unlikely
SHS1	Verified	GO:0007047 "cell wall organization and biogenesis"	YES	known true
TAF1	Verified	GO:0006325 "establishment and/or maintenance of chromatin architecture"	YES	known true
SRP1	Verified	GO:0006605 "protein targeting"	YES	known true
SPT8	Verified	GO:0016568 "chromatin modification"	YES	known true
RPA190	Verified	GO:0007046 "ribosome biogenesis"	YES	highly unlikely
NGG1	Verified	GO:0006357 "regulation of transcription from RNA polymerase II promoter"	YES	known true
RLR1	Verified	GO:0000723 "telomere maintenance"	YES	unlikely to be true
ESA1	Verified	GO:0006338 "chromatin remodeling"	YES	known true
